# Engrailed homeoproteins in visual system development

**DOI:** 10.1007/s00018-014-1776-z

**Published:** 2014-11-29

**Authors:** Andrea Wizenmann, Olivier Stettler, Kenneth L. Moya

**Affiliations:** 1 Department of Anatomy, Institute of Clinical Anatomy and Cell Analysis, University of Tübingen, Österbergstrasse 3, 72074 Tübingen, Germany; 2 Laboratoire CRRET EAC 7149, Université Paris-Est Créteil, 61, Av. du Général de Gaulle, 94010 Créteil Cedex, France; 3 Collège de France, Center for Interdisciplinary Research in Biology, UMR CNRS 7241/INSERM U1050, 11 place Marcelin Berthelot, 75005 Paris, France; 4 Labex Memolife, PSL Research University, Paris, France

**Keywords:** Visual system, Retina, Tectum, Sensory map, Homeoprotein, Engrailed

## Abstract

Engrailed is a homeoprotein transcription factor. This family of transcription factors is characterized by their DNA-binding homeodomain and some members, including Engrailed, can transfer between cells and regulate protein translation in addition to gene transcription. Engrailed is intimately involved in the development of the vertebrate visual system. Early expression of Engrailed in dorsal mesencephalon contributes to the development and organization of a visual structure, the optic tectum/superior colliculus. This structure is an important target for retinal ganglion cell axons that carry visual information from the retina. Engrailed regulates the expression of Ephrin axon guidance cues in the tectum/superior colliculus. More recently it has been reported that Engrailed itself acts as an axon guidance cue in synergy with the Ephrin system and is proposed to enhance retinal topographic precision.

## Introduction

Since the discovery of homeobox genes [1, 2] there has been accumulating evidence from all multi-cellular organisms that these genes play key roles in determining positional information. These genes encode homeoprotein transcription factors that regulate the expression of downstream genes necessary at all developmental stages, including lineage determination, cell migration, cell differentiation, and tissue formation. Some homeoproteins are also able to regulate protein translation and cell-to-cell signaling. The proteins of the Engrailed family can exert all three functions, regulate gene transcription and protein translation, and act in an extracellular signaling pathway. All three of these functions of Engrailed are put into play for the correct development of the visual system in vertebrates.

### Non-cell autonomous homeoprotein activity and visual system development

The non-cell autonomous developmental function of homeoproteins has only recently been deciphered and opens an entirely new view on developmental processes. Pax 6 is necessary for eye development in many species [3–6] and this was attributed to its cell autonomous activity. However, when the intercellular passage of Pax6 is disrupted in zebrafish embryos, fish develop dissymmetric eyes, one eye or no eye phenotypes [7]. The homeoprotein Otx2 is expressed in the retina and is important for retinal ganglion cell (RGC) prenatal specification and, after birth, for the maintenance of cone photoreceptors, bipolar cells and RGCs [8]. Since postnatal RGCs do not express Otx2, this dependence on Otx2 for maintenance or neuroprotection [9] is another example of non-cell autonomous homeoprotein activity.

Beyond the retina, Otx2 can be transferred to the visual cortex from external sources and blocking its transfer and accumulation in parvalbumin cells within layers III and IV of visual cortex regulates the opening, closure or reopening (in the adult) of a critical period for the plasticity of the visual cortex [10–12].

Engrailed proteins now have a twofold role for the development of the subcortical visual connections: first, Engrailed transcriptional activity is important for the formation of subcortical visual structures in the brain; second its protein translation and cell–cell signaling properties guide retinal axons in the formation of visual maps.

### Engrailed

The Drosophila gene *Engrailed* was first identified in 1929 as an autosomal recessive gene [13]. Since the mutant possessed a dent in the scutellum Eker called it *Engrailed* after ‘engrailé’ a heraldic term from middle-age French meaning ‘dented by hail’. *Engrailed* turned out to be a key selector gene that is involved in the development of posterior compartments of appendages and segments [14–16] and the nervous system [17, 18] during Drosophila development. Since then, one or more Engrailed proteins have been described in many metazoans from echinoderms [19], nematodes [20], annelids [21], brachiopods [22], platyhelminthes [23], molluscs [24], cephalochordates [25], onychophorans [26] priapulids [27] and in vertebrates [28]. Duplications generated several *Engrailed* paralogues in different organisms (for review see: [29]). Vertebrate homologues were discovered in chick, mice, frogs and fish [28, 30–32]. Vertebrates in general have 2–3 *Engrailed* genes and in most species they confer specific identity to defined areas and neurons.

**Fig. 1:**
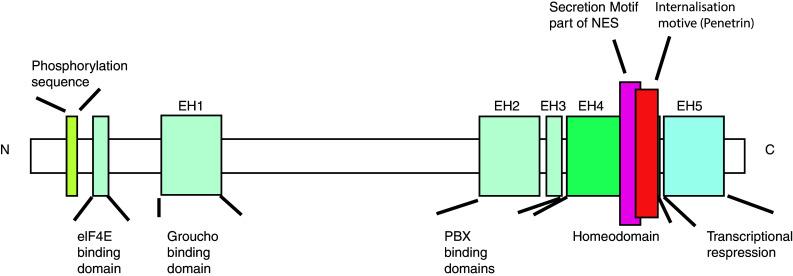
Functional domains of Engrailed proteins. En1/2 contain a classic homeodomain. Within the homeodomain, Engrailed proteins have a secretion and an internalization motif (Penetratin). At the N-terminal region a binding site for elf4E and a phosphorylation site were identified. Elf4E initiates protein translation and the phosphorylation appears to regulate En1/2s association with membrane fractions enriched in cholesterol and glycosphingolipids. NES is the nuclear transport signal within the homebox. Modified from Morgan 2006. See text and Morgan 2006 for references

Engrailed proteins contain highly conserved homeodomains (Fig. 1), domains involved in active repression of transcription [33], and domains that bind important co-factors like Groucho and Extradenticle (Exd)/Pbx [34, 35]. The phosphorylation of specific residues increases DNA binding [36]. With Exd as a cofactor Drosophila Engrailed, normally a repressor, can also act as transcriptional activator in vivo [37]. Like other homeodomain proteins Engrailed protein also acts as translational regulator and interacts with elF4E [38–40]. Surprisingly, Engrailed also possesses domains that allow the protein to be secreted and internalized [41]. That Engrailed transcription factors contain these domains and transfer between cells has been reported for some time but only recently has the physiological significance of this been fully appreciated (for reviews see [12], [42]).

### The vertebrate visual system and topographic maps

An essential aspect of nervous system development is the establishment of precise functional neuronal connections in the brain. Locally, these connections can form specific networks (i.e. a cerebral maps) that topographically reproduce the spatial organization of the peripheral sensory receptors. The development of precise projections implies that growing axons, (1) carry an identity of their place of origin; (2) follow the correct pathway towards their target; and (3) recognize a local “stop signal” to synapse on their proper target cells. The overall process requires in addition, the specification and differentiation of the target territories since the establishment of sensory connections and the development of their targets in the brain are more or less simultaneous phenomena.

One of the best-studied sensory maps is the one formed by retinal axons in the brain. In the vertebrate visual system, photoreceptors in the retina transduce light information (i.e., photons) into neuronal signals. Bipolar cells in the inner nuclear layer of the retina receive the transduced light information from photoreceptors and convey it to RGCs. The synaptic activity between photoreceptors and bipolar cells can be modulated by horizontal cells, and the synaptic activity between bipolar cells and RGCs is modulated by amacrine cells. RGC axons constitute the only efferent pathway from the retina and their terminals form visual maps in the brain.

**Fig. 2:**
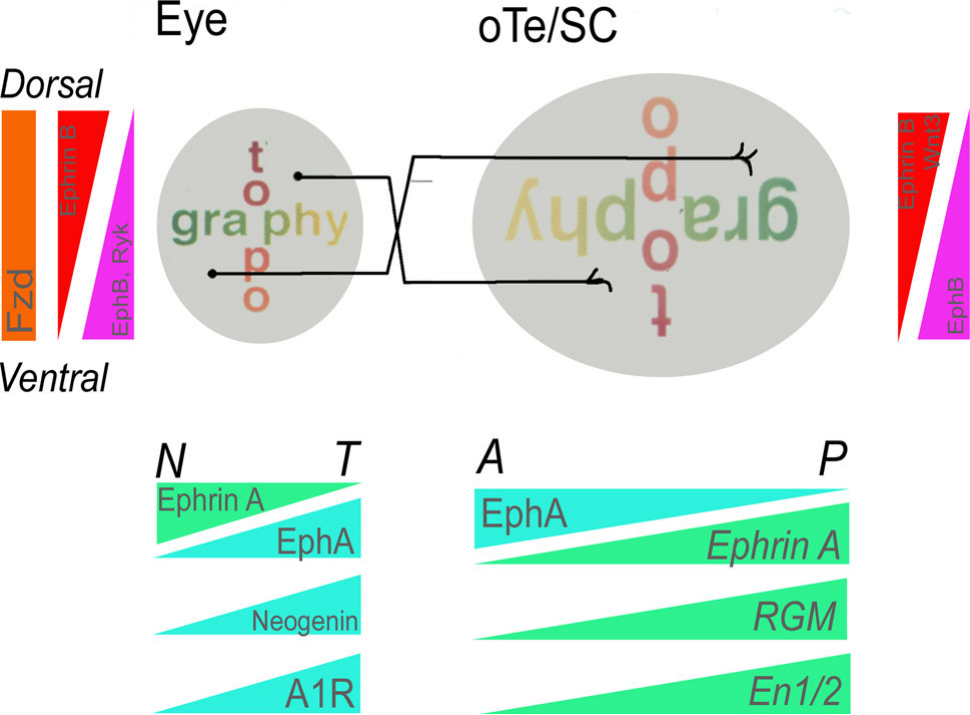
Topographical organization of the retinotectal system and the graded expression of guidance cues and receptors. Anterior tectum receives the input from temporal axons, posterior tectum receives input from nasal axons. Within the tectum the earliest graded expression is that of En1/2, which shows a high expression in the posterior and a low expression in the anterior tectum. Later different Eph As and Ephrins—the member depends on the species—are expressed in counter gradients along the tempero-nasal axis of the retina and the anterior–posterior axis of the tectum. RGM shows an anterior-to posterior raising gradient in the tectum and its receptor, Neogenin, a countergradient in the retina. Another temporal to nasal gradient is formed by the A1R receptor in the retina. Along the dorsoventral axis of retina and mediolateral in the tectum are countergradients of EphB and EphrinB as well as Wnt its receptor Frizzeled (Frz)

The retinal projection to the brain is topographic, which means that the spatial order of neuronal origin in the retina is reflected in the spatial order of their axon terminals in the target area [43]. A topographic retinal map is thus formed by RGC axons in the lateral geniculate nucleus of the thalamus, which projects the map into the visual cortex, and in the dorsal mesencephalon where RGC axons synapse in the optic tectum (oTe) as it is called in birds or the superior colliculus (SC) in mammals. The retina is represented topographically in the oTe/SC such that axons from temporal retina project to the anterior oTe/SC, and axons from nasal retina project to the posterior oTe/SC. The dorso-ventral axis of the retina is represented along the latero-medial axis of the tectum (see Fig. 2).

One early theory for how this precise map is established during development was suggested by Sperry more than 50 years ago as the ‘chemoaffinity hypothesis’. Based on eye rotation experiments in amphibians he postulated two orthogonal ‘cytochemical’ gradients in the retina, so as to impose positional identity onto each retinal ganglion cell ‘in a kind of chemical code’ along the naso-temporal and dorso-ventral axis of the eye [44]. These gradients would then be matched with complementary gradients in the tectal target field of the retinal projection (Fig. 2). Mathematical models for the arrangement of these gradients, and the capacity of growth cones to correctly navigate along these gradients were proposed by Gierer [45–47]. Subsequently, numerous studies in vivo and in vitro provided evidence for the existence of positional cues along the rostrocaudal and mediolateral axis of the tectum thought to guide both developing and regenerating retinal axons to their correct target cells [48–52].

### In vitro evidence for molecular guidance cues

At the beginning of the 80s the first molecular markers were found that displayed a graded distribution in retina and oTe (for review see: [53] In 1987, Bonhoeffer and colleagues provided the first biochemical evidence for the existence of guidance molecules as proposed by Sperry [48, 54]. They showed that the growth of retinal axons on alternating stripes of membranes from posterior and anterior tectum resulted in an invariable preference of temporal axons for anterior oTe, their natural target. The choice of temporal axons did not seem to be influenced by attractive cues from anterior membranes but rather by repulsive cues from posterior membranes. So far, in all species examined (chick, mouse, fish and rat), temporal retinal axons avoid growing on membrane stripes from the posterior oTe/SC [48, 51, 54–56].

These in vitro experiments demonstrate that the oTe expresses positional cues to which RGC axons are able to respond. When tectal vesicles were prepared under conditions that depleted them of some low molecular weight proteins and enriched them for high molecular weight cell surface proteins, nasal axons grew preferentially on posterior membranes likely due to attraction [50]. Repulsive cues were demonstrated in a different assay system in which membrane vesicles from posterior tectum caused a reversible collapse of temporal growth cones [57]. A similar collapse-inducing activity was also obtained with purified glial cells from Xenopus posterior oTe [58]. Repellent and collapse-inducing activity turned out to be identical [59]. Target-specific innervation by axons along the dorso-ventral axis of the tectum in vivo was demonstrated much later [60, 61], although in vitro experiments had already indicated that cells from dorsal retina preferentially adhere to ventral oTe/SC, and vice versa [62].

### The identification of “classical” guidance molecules

The first molecule found to influence the choice behavior of temporal axons in vitro was called ‘retinal guidance molecule a’ (RGMa); [63, 64]. However, the RGMa mutant lacked any defect in neuronal projections [65]. In 1995, two EphrinA ligands with low anterior and high posterior expression in the tectum, EphrinA5 (formerly called RAGS—repulsive axon guidance signal) and EphrinA2 [formerly called—Elf-1 (Eph ligand family)] were discovered [66, 67]. Their expression along the anterio-posterior axis in the tectum and their activities are not equivalent. EphrinA2 expression extends more anteriorly than that of EphrinA5. Both repel temporal axons [68, 69] and high concentrations of EphrinA5 also repel nasal axons [69]. Both molecules interact with the same set of EphA receptors present on RGC axons and the receptors are more abundant on temporal axons than on nasal axons [69] (Fig. 2). Additional EphA receptors and EphrinA ligands with graded and linear expressions along the AP axis of the tectum/SC and the retina have been described (for review: [70, 71]) (Fig. 2).

Thus, RGCs and oTe/SC cells each express both Eph and Ephrin proteins in complementary gradients (Fig. 2). Eph and Ephrins can interact in cis (i.e. at the same cell/axon) and in trans, can signal bidirectionally and a given Ephrin can be repulsive or attractant. These properties allow for a complex set of interactions (for review: [72]). Cis signaling regulates the sensitivity of retinal axons to Ephrins in the oTe/SC and can change the expression pattern from uniform to graded [73]. Thus, the more Ephrins an axon expresses that will interact with its Eph receptor the less sensitive it is to trans Ephrins in the tectum [73–75]. In addition, EphA3 ectodomain expressed in a decreasing anterior–posterior gradient in the oTe contributes to nasal growth cone preference for posterior oTe and thus complements the anterior-posterior EphrinA repellent gradient [76].

Subsequently, EphrinB and EphB proteins were identified as candidates for the dorso-ventral mapping labels. EphrinB1 is present in a medial high to lateral low gradient in the oTe/SC and the receptor EphB shows a ventral high and dorsal low gradient in the retina [60, 74, 77–81] (Fig. 2). Two earlier studies showed that EphB-Ephrin-B signaling accounts in part for retinotopic dorso-ventral mapping [59, 80]. EphrinB1 can act both as attractant and repellent for RGC axon side branches contributing to the precision of the medio-lateral map [82]. More recently the combinatorial contribution of multiple EphB receptors in response to EphrinB1 has been reported [82]. Using EphB1-3 null mice, McLaughlin et al. reported that while the qualitative errors in retinotopic mapping were not altered, there was an important dose effect. In other words, as more EphB alleles were silenced, the frequency of aberrant projections increased.

To complicate matters, several proteins seem to interfere with EphrinA expression in the retina and thus in the retinotopic map formation. Ventroptin, a BMP-4 antagonist, is necessary for correct Ephrin expression in the retina [83]. Several neurotrophins (p75, proBDNF) appear necessary for the repellent effect of EphA receptor on nasal axons [84, 85].

Similar to Ephrin-B expression is Wnt3 expression with a medial high to lateral low gradient and its receptor Ryk is present in a ventral high and dorsal low retinal gradient [61]. Wnt/Ryk also play a role in visual system axon guidance. The Ryk receptor mediates repulsion and Frizzeled receptors mediate attraction at low levels of Wnt3 [61]. These properties allow for a baffling set of interactions influencing the establishment of retinotectal projections (review: [72, 86]).

The Ephrin expression pattern corresponds well with Sperry’s chemoaffinity hypothesis [44]. And, Eph/Ephrin signaling is important for visual map formation. Loss of EphrinA5 and EphrinA2 in the mouse results in topographic errors of retinal axons in the SC [87, 88]. The Ephrins involved in establishing the retinocollicular map are not completely redundant in their function since topographic errors are enhanced in Ephrin-A2/A5 knock-out mice [88] and Ephrin-A2/A3/A5 triple knock-out mice [89]. Although Ephrin-A2/A3/A5 triple knock-out mice have a severe mapping defect in SC and lateral geniculate nucleus, a rough topography remains. Intrinsic optical imaging [90] revealed, in knock-out mice, areas of the SC with topographically inappropriate functional responses, albeit the general polarity of the map is still functionally preserved (Fig. 2). Thus, it seems that Eph/Ephrin signaling is necessary but not sufficient to establish a complete retinotectal map. Other signaling mechanisms might, thus be involved in the creation of the retinal map formation in the oTe/SC. Recent reports showed that Engrailed proteins play a role in guiding retinal axons along the oTe in a non-cell autonomous manner.

### Engrailed contributes to the formation of the retinorecipient mesencephalon

The oTe/SC develops from dorsal midbrain and displays very early a rostrocaudal polarity. The earliest known markers for midbrain polarity are *Engrailed* genes and proteins in vertebrates. *En1* and *En2* are expressed in posterior midbrain and anterior hindbrain comprising the mid-hindbrain boundary (MHB) from the mid-neural plate stage [28, 91, 92] Early on *En1* expression covers the entire mesencephalon but then declines in anterior oTe/SC so that high expression remains just anterior and posterior to the MHB. *En2* expression in mouse and chick lags slightly behind that of *En1* and persists longer in some regions like the mesencephalon [92–94] (Fig. 2).

The morphological analysis of *En* mutants and the overexpression of *En* in chick strongly suggests that En is necessary for the establishment of mesencephalic polarity. Mice homozygous for a targeted *En1* homeogene deletion die at birth and display a severe disruption of the mid-hindbrain region, among other defects [95]. Mice homozygous for a targeted deletion of *En2* show a 30 % reduction in cerebellar size and a distinct abnormality in patterning of cerebellar folds, but an apparently normal dorsal mesencephalon [96, 97]. *En1/En2* double mutants exhibit a more severe deletion of mes/metencephalic tissue than the single knockouts, which might suggest synergistic or additive effects [98]. The different phenotypes do not reflect a divergence in the biochemical activity of these two genes, but rather differences in their temporal and spatial expression patterns [99]. The *En1* mutant can be completely rescued by insertion of mouse *En2* coding sequence into the *En1* locus [99].

The oTe/SC develops from dorsal midbrain, the alar plate and very early displays a rostrocaudal polarity in its *En* expression and later in its cytogenesis and retinal innervation. Engrailed proteins are strongly expressed caudally and the rostral part of the oTe that develops earlier, shows a more advanced laminar structure and is the target of temporal axons [100, 101]. The different cytological development becomes obvious at around embryonic day (E) 5 in chick [100, 101]. A day later the first retinal axons enter the anterior oTe in the chick. Reversal of the rostrocaudal axis of the alar plate/oTe before HH stage13 [~embryonic day (E) 2] resulted in a normal i.e. anterior-to-posterior Engrailed gradient, normally developed tecta and a normal retinal projection. Thus, the reversed alar plate developed according to its new orientation by adopting the typical gene expression, histological development and retinal innervation pattern [102–105]. Tectal development and gene expression did not adapt to the host pattern when the reversal of the alar plate took place after HH14. This resulted in a strong *Engrailed* expression in anterior oTe, a delayed layering compared to the posterior end, and temporal axons never entering these inversed tecta [104]. These experiments suggested that the tectal region with strong *En1/2* expression will become posterior tectum with delayed lamination and nasal retinal innervation.

Further experiments supported that hypothesis. Misexpression of *En1/2* in chick diencephalon revealed that *En* is essential for tectal identity [106]. Ectopic expression of *En* in the dorsal diencephalon led to a rostral shift of the di-mesencephalon boundary including tectal specific markers (*Pax7*, *EphrinA2*), and changes in histoarchitecture and size of the tissue. This was not the case when *En1/2* was overexpressed in the hindbrain. The difference between di-, mesencephalon and rhombencephalon is the lack of Otx2 in the latter. Thus, it seems that without the presence of Otx2 Engrailed seems unable to induce tectal structures. Very recent results suggest that Engrailed 2 is also important for migration and positioning of cells during tectal laminar formation [107].

To test a direct link between *En* expression and the formation of the retinotectal map in vivo, *Engrailed* was ectopically expressed throughout the tectum by introducing a replication-competent virus, encoding chick *En1* or *En2* [108, 109]. The scattered *En* expression throughout the entire tectum caused a perturbation of the retinotectal order in both studies. Nasal retinal fibers that normally arborize in the posterior SC that has high En protein level, arborized in the areas of high En protein in the anterior SC. Temporal fibers, whose natural target is the anterior SC failed to innervate the SC or degenerated. This suggests that *En* overexpression causes a local posteriorization of the anterior SC. Friedmann and O’Leary also reported that nasal retinal axons occasionally formed tight foci around *En* overexpressing cells, which might corroborate earlier in vitro findings of attractive cues that are elicited from posterior tectum [108].

Taken together *En* seems to be upstream of the repulsive and perhaps also the attractive guidance cues. Two studies tackled this question in vivo by a virally directed mis-expression of mouse *En1* or *En2* in the chick midbrain [94, 110]. They show that *En1*-infected anterior oTe repels temporal axons in the stripe assay. This repulsion could be correlated with an ectopic expression of *EphrinA2* and *A5*, which are upregulated in the anterior oTe as a consequence of *En1* overexpression. In addition, the normal cytoarchitectural gradient of the dorsal mesencephalon was delayed in places with ectopic *En*.

En is mostly a transcriptional repressor and therefore the induction is presumably indirect. This was supported by the observation that EphrinA2 and A5 were not always found near ectopic En expression sites [94]. The induction of ectopic EphrinA2 and A5 expression by Engrailed proteins was restricted to the mesencephalon [110] and may require Otx2 (see above). Not only *Engrailed* but also the paired box gene *Pax7* might be upstream of *Ephrins*, as a study by Thomas et al. [111] suggested. However, so far no one has determined whether the *Pax7* knockout lacks *Ephrin* expression in the SC.

Continued *Engrailed* expression may not be necessary for retinotectal map formation. Retaux and Harris [112] used an *En1/En2* antisense (AS) oligonucleotide approach to inhibit *En* expression after mesencephalic neuroepithelium was specified but before the retinotectal projection developed. In this experimental paradigm RGC axons were still able to find their appropriate topographic location within the tectum. This indicates that early *En* expression is sufficient to establish the complete tectal map and suggests that Engrailed transcription factors regulate the expression of guidance cues that are responsible for patterning retinal axon terminals in the dorsal midbrain.

### Engrailed regulation in retinorecipient midbrain

Engrailed proteins are very early positioned along the MHB even before it is established. *En1* is expressed before *En2* and shows a steeper gradient than *En2* (Fig. 2). Experiments in mouse suggested that a signal from anterior notochord activates *En1* at the same time as *Wnt1* [113]. A transient *Fgf4* expression in anterior notochord seems to be responsible for the induction of *En1* in chick [113]. However, *Fgf4* is not present in the notochord of other species, although it is conceivable that different Fgfs perform this function in other species.

The initiation of *En1/2* expression is followed by a so-called maintenance phase, in which Fgf8, Wnt1, Pax2/5/8 and En1/2 maintain each other’s expression [114]. In Zebrafish early *En1/2* expression has been shown to depend on a correct Pax2 function [115]. The continued interaction of En1/2 and the other early proteins around the notochord is mirrored in the different knock outs. The loss of Pax2, Pax5, Fgf8 or Wnt1 function allows the induction of *En1/2* genes but not their maintained expression [95, 115–119]. The interactions between these maybe indirect or even possibly recursive. Thus, in Xenopus the *En* promoter contains functional Tcf binding sites (McCrew 1999) [120] while *En1* regulates Wnt1 expression indirectly via Tcf4 [121].

Both, En1 and En2 proteins are expressed as gradients in the midbrain whereby the En1 gradient begins more posteriorly than En2 gradient and is also steeper. The graded distribution has been shown to depend on Greg4 and Fgf8 [122, 123] Fgf8 proteins are secreted and show a long-range anterior low and posterior high-graded expression along the midbrain [122]. Chen et al. [122] also revealed that different Fgf concentrations can instruct graded *En2* upregulation in vitro. Grg4, a transcriptional activator that is expressed in a countergradient across the mesencephalon, downregulates *En1/2* and *Pax5* expression. At the same time Greg4 initiates *Pax6* expression and thus promotes diencephalic development [123]. In contrast *En1/2* overexpression in the diencephalon initiated midbrain development [109]. Thus, *En1/2* are sufficient for midbrain initiation in the Otx2 expressing forebrain (see above). Zic1, an early transcription factor was also able to expand the expression of *En2* indirectly via activation of *Wnt1*. Whereas Zic1 antimorph protein inhibited Wnt1 and En2 protein expression [124].

The precise spatio-temporal expression of genes within the midbrain and the orthologues involved vary between vertebrate species [125]. However, their interactions result in stable and graded *En1/2* expression within the midbrain.

### Engrailed in invertebrates

Invertebrates require Engrailed for the formation and organization of several neural systems. In drosophila, correct *En* expression is required for normal development of midline motor and sensory pathways, as well as for synaptic connection specificity of auditory neurons [126, 127]. In the cockroach, Engrailed has been shown to play a direct role in sensory axon guidance, target recognition and terminal branch morphology [128, 129]. With regards to the invertebrate visual system, Engrailed is involved more in eye/ocelli development than brain structures.

In most invertebrates the visual system develops from the protocerebrum and the eye/antennal disc. *En* is expressed bilaterally at the posterior border of the developing protocerebrum in insects, crustacean and myriapods and forms the so-called ‘head spots’ after Roger and Kaufmann [130]. In *Drosophila melanogaster* ‘the head spot cells’ lose *En* expression when neuroblasts delaminate from ectoderm. Some of these neuronal derivatives begin to express *En* again when they form the so-called secondary head spots [131, 132]. The developmental origin of the secondary head spots is different between the various studied insects ([130–134], for review see: [135]). It is currently unclear if these cell clusters represent a homologous group throughout Insecta. A single cell analysis of secondary head spots in grasshoppers by Boyan and Williams [131] revealed that these cells contribute to the primary axon scaffold in the embryonic grasshopper brain. They project their axons into the optic tract towards the median brain in grasshopper and marbled crayfish  [131, 136].

In *Drosophila melanogaster* Engrailed is also found downstream of orthodenticle (otd) during eye formation [137] and together with sonic hedgehog plays a role in the formation of the median ocelli [138]. The eye of the *Onychophora euperipatoides kanangrensis* is homologous to insect ocelli. That the ocelli also express Engrailed [139] supports a homology of ocelli between these species. However, the spider *Cupiennius salei* shows *En* expression at the site of the posterior median eye, which is not truly homologous to the median ocelli of insects [140]. This difference in expression indicates a different role of Engrailed during eye formation in the spider. Thus, in invertebrates Engrailed is present in eye/ocelli and plays a role in their development and this is in contrast with vertebrates, in which Engrailed is not expressed in the eye or retina. Another difference is that while in invertebrates Engrailed is only known to act as a transcription factor, in vertebrates, Engrailed can have other activities (see below).

### Engrailed as signaling factor in the vertebrate primary visual system

In vertebrates Engrailed can also transfer between cells and has non-cell autonomous activities [141]. In a turning assay of Xenopus retinal explants in culture, a gradient of exogenous En2 attracts nasal RGC axons and repels temporal RGC axons [40]. This RGC axon guidance activity required the internalization of En2 by the growing axons and is dependent on local protein synthesis independent of the cell body. When we examined the chick oTe, we found that 5 % of En1/2 proteins are associated with the extracellular side of tectal membranes and are present in a low anterior and high posterior expression gradient [142]. Interfering with the transfer of extracellular Engrailed in vivo in Xenopus and chick oTe led to an abnormal retinotopic map formation where temporal RGC axons grew into posterior parts of the oTe [142]. While Eph/Ephrins have been shown to function as rough guidance molecules, low physiological concentrations of Engrailed sensitized temporal RGC axons to repulsive effects of very low concentrations of EphrinA5 that on their own do not repel temporal axons.

### Engrailed signals through mitochondrial activation and adenosine

Recently, we characterized the non-cell autonomous engrailed signaling pathway in axon guidance. Using metabolic labeling of growth cone particles prepared from embryonic mouse SC, we observed an eightfold increase in the neosynthesis of Ndufs3, a key component for the assembly of complex I of the mitochondrion [143]. This led us to hypothesize that perhaps Engrailed increased mitochondrial activity in growth cones. Indeed, exogenous Engrailed produced a rapid neosynthesis and release of ATP from growth cones.

NADPH fluorescence was used to visualize and quantify extracellular ATP and we observed an increased fluorescence at the growth cone within 1–5 min after the addition of Engrailed to the culture medium. This ATP response varied in timing and intensity from growth cone to growth cone but the peak response was about 100 s after the onset of ATP release [143]. Pretreatment with anisomycin, a protein synthesis inhibitor strongly inhibited the release of ATP after Engrailed. A mutant form of Engrailed that retains its transcriptional activity but that is defective for binding eIF4E did not stimulate synthesis and release of ATP when added to the growth cones. Taken together, this series of experiments demonstrated that extracellular Engrailed induces a rapid and protein translation-dependent ATP synthesis and release by RGC growth cones.

The growth cone collapse assay was used to dissect the extracellular ATP signaling pathway of Engrailed [143]. In these experiments EphrinA5 at a concentration of 0.1 µg/ml increases collapse frequency from 8 to 24 % compared to the maximal 50 % value obtained with 0.4 µg/ml. Engrailed alone at a concentration of 75 nM had no effect but raised the frequency of growth cone collapse to 41 % in the presence of weak 0.1 µg/ml of EphrinA5. The protein synthesis inhibitor anisomycin only blocked the latter Engrailed synergizing activity. Thus, EphrinA5-induced collapse is not protein synthesis dependent while Engrailed synergistic collapse activity is protein synthesis dependent.

**Fig. 3:**
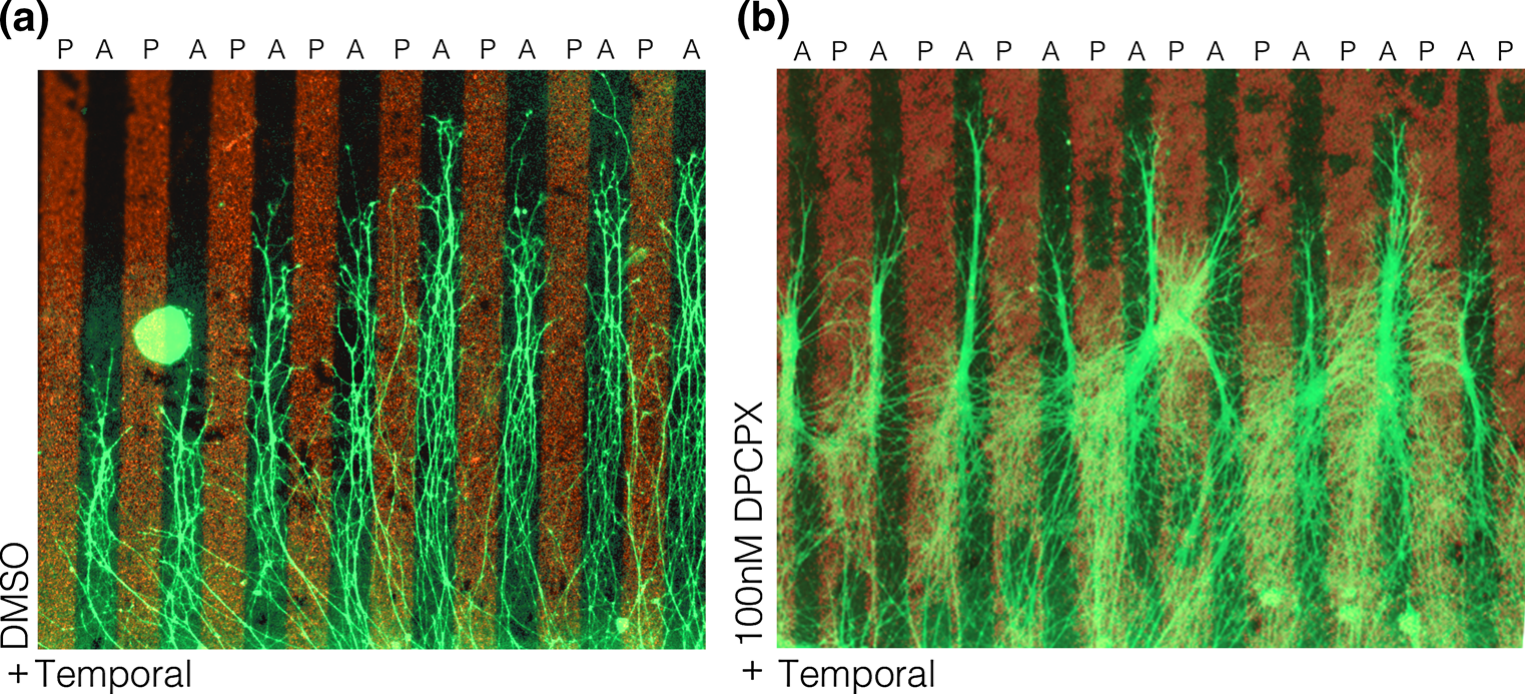
A1R is involved in repelling temporal axons from posterior membranes. **a** Temporal retinal axons prefer to grow on membranes from anterior tectum when given the choice between anterior and posterior (indicated with *red fluorescent beads*) membranes. Nasal Axons grow on both types of membranes (not shown). **b** Adding the A1R-specific antagonist DPCPX reduced the effect of posterior membranes on temporal axons. Many temporal axons now cross posterior membranes when the antagonist is present in the medium. Note that after DPCPX loses is activity to block A1R, temporal axons are sensitive to inhibitory cues of the posterior membrane borders again and grow on anterior membrane stripes

**Fig. 4:**
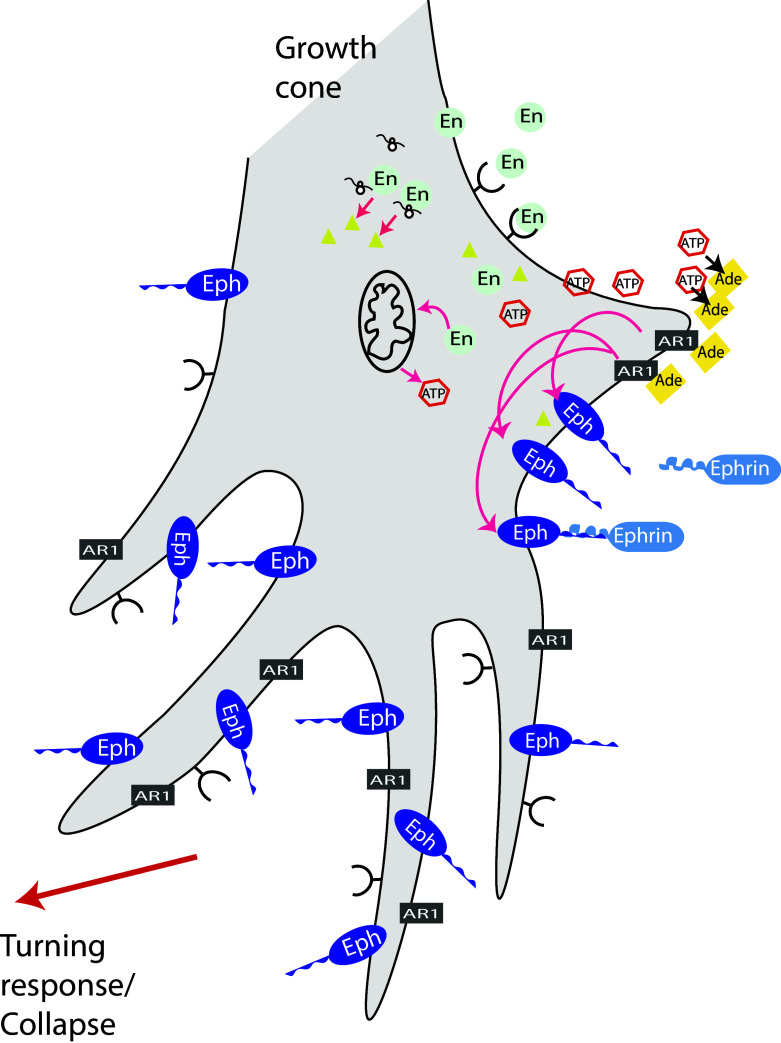
En signaling at the growth cone. En is internalized by the growth cone. Within the growth cone it stimulates translation of Ndufs3, a protein of complex I of mitochondria. This activates ATP synthesis, which in turn is externalized into the extracellular space and there it is hydrolyzed to adenosine. Adenosine activates the A1R receptor, which acts synergistically with Ephrin signaling perhaps via adenylate cyclase to cause growth cone collapse and arrest in the oTe/SC. Figure based on [143]. See text for references

When extracellular ATP hydrolysis was inhibited Engrailed-stimulated collapse was blocked, while increasing hydrolysis increased collapse [143]. Pharmacological studies further demonstrated that adenosine is the effector molecule for Engrailed and that this purine acts at the adenosine 1 receptor (A1R) on growth cones. (Figures 3, 4). In summary, Engrailed enters the growth cone and rapidly (within 1–2 min) stimulates ATP synthesis and release that is dependent on protein synthesis. Extracellular ATP is hydrolyzed to adenosine that acts at the A1R receptor in synergy with Eph/EprinA5 on the growth cone.

The experiment depicted in Fig. 3 is a classical stripe assay with an explant from temporal retina confronted with stripes of posterior oTe membrane (red stripes) or stripes of anterior oTe membranes (black stripes). In control conditions temporal retinal axons (green fibers) are repelled by posterior oTe membranes and preferentially grow on the anterior membrane stripes. After a single application of an A1R antagonist (DPCPX) soon after starting the explant culture, temporal retinal axons become insensitive to repellent cues of posterior oTe membranes. However, when the effects of the A1R antagonist wear off, the temporal axons are again repelled by the posterior oTe membranes and avoid the red stripes.

Growth cones from chick nasal retina were insensitive to Engrailed, i.e., exogenous protein did not have collapse-inducing activity. Immunofluorescence studies revealed that growth cones from temporal retina had more A1R compared to growth cones from nasal retina. This likely explains the different sensitivity of nasal and temporal growth cones to Engrailed activity.

In the chick, Engrailed does not directly control growth cone pathfinding but rather indirectly by potentiating Ephrin-A5 signaling. Thus, in some species, Engrailed may act as a modulator of Ephrins, enhancing their capacity to contribute to precise retino-tectal topographic mapping via interaction with their Eph receptors. In other species however, a more direct function of Engrailed is conceivable. In the mouse, but not in the chick, retinal growth cones could respond to low concentrations of Engrailed in absence of exogenous Ephrins (Stettler, Moya, unpublished observations). Interestingly, in the mouse, the range of concentrations of Engrailed that induces retinal growth cones collapse in vitro is lower for temporal than nasal growth cones suggesting that a temporal-nasal selectivity could be directly controlled by Engrailed itself in this species. The preservation of some polarity within the map of double and triple Ephrin knock out is thus consistent with a role of Engrailed as a direct contributor/co-guidance factor of the map formation together with an accessory function for controlling the map precision through a physiological interaction with Ephrins.

### Redundancy and synergy in the system

In vitro assays, while useful and easy to use, may not accurately mirror the in vivo situation. For example, concentrations of guidance molecules with observable effects in culture might well be above physiological concentrations in situ. In vivo then, low concentrations of guidance molecules that alone do not have an observable effect may function in concert with other molecules to ensure the precision of the sensory map. The large number of Ephs/Ephrins and other guidance molecules and molecular modulators such as Engrailed would be consistent with this idea. Not only would this provide a high level of complexity in precision patterning in the brain, but this would also build redundancy into the system.

We combined our findings to develop a computational model [141]. This model incorporated three gradients and a non-linear response of the growth cone to Engrailed. The gradients were: EphrinA5 in the tectum low anterior, high posterior; Eph on RGC axons low nasal, high temporal; Engrailed in the tectum low anterior, high posterior; A1R on the RGC axons low temporal, high nasal. The model is consistent with the observation that Eph/Ephrin signaling is sufficient for a crude map to form. However, the inclusion of Engrailed and A1R greatly enhances the precision of the retinotopic map. Interestingly, if the temporal/nasal differences in the A1R are eliminated, rather than altering the precision of the map, the model predicts that the map would be compressed in the anterior part of the tectum but would retain its high precision. It will be of great interest to test the prediction of the model in mice with modified expression of A1R.

## Conclusion

The homeoprotein transcription factor Engrailed contributes to the development of the visual system development in vertebrates in invertebrates. While it is involved in eye development in invertebrates, Engrailed influences the development of visual structures in vertebrates in several ways. Early in brain development, Engrailed acts as a classical transcription factor in conjunction with other factors to regulate the organization and establish tissue polarity of the visual dorsal mesencephalon. At later times Engrailed regulates the graded expression of classical axon guidance cues in a cell autonomous manner. Engrailed also has the unexpected capacity to be secreted from one cell and internalized by a neighboring cell. Recent studies now show that Engrailed can act non-cell autonomously to directly contribute to the formation of retinal topography in the dorsal mesencephalon. Engrailed is internalized by RGC growth cones in which it can stimulate protein synthesis and increase mitochondrial complex I activity and ATP synthesis within minutes. The engrailed-stimulated ATP is rapidly externalized where it is hydrolyzed to adenosine. Adenosine acting at the A1R then enhances growth cone collapse in response to EphrinA4. Thus Engrailed signals via ATP to render RGC axons more sensitive to guidance cues. Computational modeling confirms that this pathway, Engrailed-ATP-A1R-Ephrin, might serve to increase the precision of the retinotectal map.
